# Relationship Between Cognitive Abilities and Lower-Limb Movements: Can Analyzing Gait Parameters and Movements Help Detect Dementia? A Systematic Review

**DOI:** 10.3390/s25030813

**Published:** 2025-01-29

**Authors:** Swapno Aditya, Lucy Armitage, Adam Clarke, Victoria Traynor, Evangelos Pappas, Thanaporn Kanchanawong, Winson Chiu-Chun Lee

**Affiliations:** 1 School of Mechanical, Material, Mechatronics and Biomedical Engineering, University of Wollongong, Wollongong 2522, Australia; ssa439@uowmail.edu.au (S.A.); alucy@uow.edu.au (L.A.); 2 Advanced Mechatronics and Biomedical Engineering Research Group, University of Wollongong, Wollongong 2522, Australia; 3 School of Psychology, University of Wollongong, Wollongong 2522, Australia; aclarke@uow.edu.au; 4 University of the Sunshine Coast Sunshine Coast 4560, Australia and Warrigal, Illawarra 2527, Australia; vtraynor@usc.edu.au; 5 School of Health and Biomedical Sciences, RMIT University, Melbourne 3001, Australia; evangelos.pappas@rmit.edu.au; 6 School of Computer Science and Information Technology, University of Wollongong, Wollongong 2522, Australia; tk386@uowmail.edu.au

**Keywords:** gait, cognitive impairment, brain activity, dual task

## Abstract

Identifying and diagnosing cognitive impairment remains challenging. Some diagnostic procedures are invasive, expensive, and not always accurate. Meanwhile, evidence suggests that cognitive impairment is associated with changes in gait parameters. Certain gait parameters manifesting differences between people with and without cognitive impairment are more pronounced when adding a secondary task (dual-task scenario). In this systematic review, the capability of gait analysis to identify cognitive impairment is investigated. Twenty-three studies published between 2014 and 2024 met the inclusion criteria. A significantly lower gait speed and cadence as well as higher gait variability were found in people with mild cognitive impairment (MCI) and/or dementia, compared with the group with no cognitive impairment. While dual tasks appeared to amplify the differences between the two populations, the type of secondary tasks (e.g., calculations and recalling phone numbers) had an effect on gait changes. The activity and volume of different brain regions were also different between the two populations during walking. In conclusion, while this systematic review supported the potential of using gait analysis to identify cognitive impairment, there are a number of parameters researchers need to consider such as gait variables to be studied, types of dual tasks, and analysis of brain changes while performing the movement tasks.

## 1. Introduction

Dementia or cognitive impairment can be caused by various diseases gradually affecting memory, thinking, and the ability to carry out day-to-day activities [1]. People with dementia mostly comprise adults over 60 years old with the likelihood of dementia increasing significantly with progressive aging [2]. Dementia gradually impacts movement and mobility, speech, memory, and cognition [1]. For example, people living with dementia, due to their changes in cognition, are often shown to experience slow and irregular movements [3], which pose a significant risk of falling while walking [4,5]. Falls hinder the quality of life in this population as well as in older adults more broadly, potentially exposing them to fractures that may lead to increasing death risk [6].

Early diagnosis is crucial in improving the quality of life for people with dementia. It facilitates early planning for the person’s cognition for as long as possible and to make necessary adjustments and preparations for the future [7,8]. However, diagnostic methods have limitations. For example, neuroimaging (MRI, CT scans), spectroscopy procedures, and laboratory tests are usually expensive and time-consuming [9,10]. Paper-based cognitive assessments rely on questionnaires that aim to test working memory and understanding of shapes and patterns as well as computation skills, which may be biased against people with language barriers and low levels of educational attainments [11]. Many tests are still to be translated and adapted to different languages; some questions and instructions in these tests are difficult to explain or translate in certain languages [11]. One alternative could be to analyze gait patterns in different conditions. Gait analysis involves equipment that is cost-friendly compared to neuroimaging, and it just requires the patients to walk.

Gait is a candidate motor biomarker of cognitive impairment and dementia [12,13]. A significant decrease in brain functional connectivity combined with a decreased integrity in white and gray matter exists among people with dementia [14,15]. Such decreases cause a reduction in mobility [15]. Deterioration of gait can occur because it involves multiple processes from sensory integration to movement execution and relies heavily on the cortical and subcortical parts of the brain and spinal cord [16]. Changes in these parts of the brain, as seen in dementia, can hinder proper movement execution, thus further hindering the normal gait cycle. Furthermore, executive functions of the brain, which are mostly responsible for cognition, working memory, and so on, have a direct correlation to changes in certain gait parameters such as velocity, step length, motion, etc. [17].

Gait speed changes have been identified in persistent cognitive impairment, which hints towards early-stage or pre-clinical dementia [18]. However, gait speed may be an overly simplistic cognition marker, especially if participants possess a certain level of physical fitness combined with a mild case of cognitive impairment [19,20]. Changes in other gait parameters, such as higher stride time and stride variability, were also noticed among people with dementia, cognitive impairment, and older adults [21,22].

Dual tasks involving the analysis of movement tasks (e.g., walking) while the participants perform a secondary task (e.g., mathematical operations) were also used to explore human cognitive capabilities. There were more prominent differences between adults with and without cognitive impairment in dual tasks, especially with cognitive-based tasks, compared to single tasks [23,24,25]. Older adults prioritize cognitive tasks over walking tasks during dual-task walking, which increases their fall risks significantly [26]. Dual-task costs, which refers to the effort to maintain stability in dual-task performance, were found to be much higher for people with dementia [27]. Considering how gait parameters are directly impacted due to cognitive strain and shared resource allocation resulting from dual tasking, this protocol has the potential to become a useful predictive tool towards isolating the early diagnosis of MCI (mild cognitive impairment) and/or dementia.

EEG studies have been combined with dual-task walking protocols to understand better the changes occurring in the brain activity in the event of multitasking, specifically cognitive resource allocation. Activation of the prefrontal cortex (PFC) of the brain is a common phenomenon observed during dual-task walking studies [28]. Protzak and Gramann [29] investigated the modulations of the alpha and beta band using EEG during motor dual-task walking (walking while pressing buttons) and discovered that alpha and beta desynchronization was less among older individuals compared to younger individuals. This lesser reduction in beta desynchronization was associated with a slower gait speed while dual-task walking, which may indicate an imbalance in cognitive resource allocation among the elderly. Thus, it is a practical approach to observe brain activity and how power and volume distribution changes across the brain during dual tasking, to understand how cognitive resources are stressed.

The main aim of this systematic review is to understand whether single-task (involving analysis of movement only) and dual-task (involving analysis of movement while performing a secondary task) protocols can identify differences between people with no cognitive impairment, MCI, or dementia. In this systematic review, we aim to identify (1) those gait parameters that are particularly useful in detecting MCI or dementia, (2) differences between single- and dual-task protocols, (3) effects of the type of secondary task type used in dual-task protocols in identifying cognitive impairments, and (4) brain activities while performing movement tasks. A systematic review is selected over other methods of review as it provides a clear and comprehensive outline of what has already been found in the current literature, and it can assist in identifying any underlying research gaps, which can lead to potential future research directions. A systematic review also enables the authors to clearly identify the methodologies that have been implemented and interesting results that have been achieved regarding this topic. This provides insight into what approaches and methodologies show promise, and which methodologies have not worked. This information can also assist in the setup and design of a research study without the possibility of replicating already published methods or running ineffective experiments. It should be noted that the target population of the studies reviewed in this paper are older adults suffering from MCI or dementia. Studies looking at stroke and other neuromusculoskeletal diseases have not been included in this review

## 2. Materials and Methods

### 2.1. Literature Search

The ‘PRISMA’ guideline was utilized for the search of the relevant literature for the purpose of this systematic review. The methodology of a literature search has been widely used in many credible review studies [30,31]. The 2020 version of the PRISMA guidelines was utilized for the literature search and screening process. The literature search was conducted through the following three databases: PubMed, Science direct, and Web of Science for January 2014–December 2024. This search duration was comparable to other review studies in this field [32,33] (10–12-year span). The following keywords were used: (a) gait; (b) dual-task; (c) MCI or dementia. Further inclusion and exclusion criteria were applied, as reported in Section 2.3.

This study involved searching for studies that involved older adults without cognitive impairment and older adults with MCI or dementia.

### 2.2. Inclusion and Exclusion Criteria

Inclusion criteria for the search included the following:

- Studies focused on either gait or lower-limb movement in cross-sectional studies.
- Studies involving people with dementia or mild cognitive impairment.
- Full-text articles.
- Studies that investigated single and dual tasks and their outcomes on people with MCI or dementia.

Exclusion criteria for the search included the following:

- Studies focused on upper-limb movements.
- Studies examining the impacts of drugs or alcohol.
- Studies not published in the English language.
- Studies not observing dual-task protocol.
- Studies observing Parkinson’s or other neurodegenerative diseases.

### 2.3. Data Extraction

The following PRISMA 2020 flow diagram for systematic reviews, which included searches of databases and registers, revealed how the studies were filtered. The initial search using the keywords “Dual task” AND “Gait” AND “Mild cognitive impairment” OR “Dementia” found a total of 3563 records across PubMed (n = 1178), Science Direct (n = 748), and Web of Science (n = 1671). Following the criteria, which include cross-sectional studies looking into gait or lower-limb movements only and removing systematic reviews, a total number of 971 studies (Pubmed n = 230, ScienceDirect n = 661, Web of Science n = 80) remained. Further exclusions were attributed to duplicate papers (n = 15), studies focusing on upper-limb movement (n = 62), studies focusing on other neurodegenerative diseases (e.g., Parkinsons, multiple sclerosis, n = 157), studies not published in English (n = 12), studies with no dual-task protocol (n = 111), studies only focused on dementia assessment and prevention (n = 187), and other topics such as exercise outcomes, balance, or assessment results (n = 358). After further screening, another 44 studies (9 studies not retrieved, irrelevant participation = 15, and irrelevant study focus = 20 studies) were excluded. This left a selection of 25 studies. Two more studies were excluded after a final scanning of the abstracts, due to not having MCI or dementia participants. This left a final list of 23 studies to be added within the systematic review.

### 2.4. Quality Assessment of Studies Retrieved

**Figure 1:**
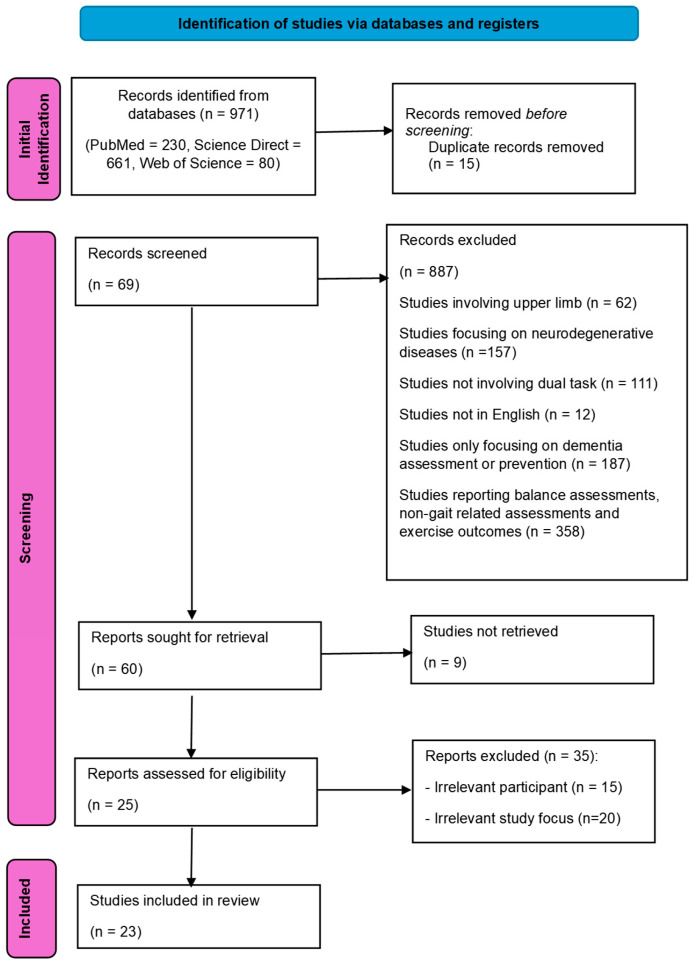
PRISMA 2020 flowchart for literature search and review.

The studies retrieved through the forementioned (Figure 1) screening process were assessed for validation, prior to including them in the review. Two reviewers independently assessed the quality of the retrieved studies, using the scoring protocol developed by the Oxford Center for Evidence-based Medicine [34]. This particular scoring protocol utilized a grade of recommendation along with a level of evidence for each study. This scale includes 10 levels of evidence, which is split into 4 grades used for recommendation (Please see Table 1 and Table 2).

## 3. Results

A total of 23 studies met the inclusion criteria for the purpose of this systematic review. After the quality assessment process, all of the 23 studies had an evidence level of either 2b or 3b and a recommendation level B according to the Oxford Center for Evidence-based Medicine—March 2009 [34]. All the studies included older adults (50+ years old) as participants, with a minimum sample size as small as n = 12 and a maximum sample size of up to n = 560. Table 3 summarizes the key highlights of the 23 studies. It can be noted that most studies report notable differences in gait parameters between older adults with no cognitive impairment and older adults with cognitive impairment. None of the studies mentioned if the participants were indigenous or from First Nation communities.

### 3.1. Participant Groups, Use of Sensors, and Test Conditions

All participants in the reviewed studies were above 50 years old (majority being over 60), who were recruited through the community, research institutes associated with universities, hospitals, and age care facilities. MCI was confirmed in these reviewed studies by assessments such as MoCA (Montreal Cognitive Assessment) [58], MMSE (Mini Mental State Exam) [59], and Petersen’s criteria [43]. Participants with dementia had their diagnosis confirmed through assessments such as Diagnosis and Statistical Manual of Mental Disorders (DSM-V TR) [39], Clinical Dementia Rating (greater than 1), and professional evaluations performed by expert clinicians [39]. One study [57] evaluated potential or subjective cognitive impairment based on self-report on declining memory loss or concerned about memory loss.

One study used 3-dimensional motion tracking cameras with reflective markers attached to the bodies [45]. Some used wearable acceleration sensors, together with the use of a traditional stopwatch to measure gait speed [36,39,41,42,52,54]. A GaitRite^TM^ walkway system and vicon nexus motion tracking camera system were also used in a number of studies measuring spatiotemporal parameters [34,44,48,56,57]. For measuring brain activity and volume, FNIRS (Functional Near-Infrared Spectroscopy) and MRI (Magnetic Resonance Imaging) were the most common equipment used [39,41,51,56].

In total, 85% of the studies reviewed in this systematic review conducted both single-task and dual-task experiments involving a control group with no cognitive impairment and a group with MCI and/or dementia.

### 3.2. Gait Parameters Comparison for Single and Dual Tasks

In all reviewed studies, single tasks involved participants walking at a self-selected comfortable walking speed on a level surface. Dual-task protocols usually consisted of a motor task (usually gait) and a cognitive task. There were also examples of motor–motor-based dual tasks in which participants walked while conducting a secondary movement task. Gait parameters associated with single and dual tasks were measured and compared in most studies, and included parameters such as gait speed, step time and length, stride time, stride to stride variability, swing time, and stride frequency.

#### 3.2.1. Gait Speed

As presented in Table 4, seven studies [37,42,44,45,55,56,57] reported that people with MCI and/or dementia had a notably lower walking speed when compared to the group with no cognitive impairment in both single- and dual-task conditions (not all studies showed significant differences between groups). However, care should be taken when interpreting the results as these studies compared different participant groups. Four of these studies [37,44,45,56] investigated differences between control groups with no cognitive impairment and groups with MCI only, which reported that MCI groups walked notably slower in both single and dual tasks. Meanwhile, the other three studies analyzed group differences among controls with no cognitive impairment and MCIs, as well as people with mild dementia and subjective cognitive impairment. Konig et al. [42] reported that people with dementia walked significantly longer over the same walking distance compared to the group with no cognitive impairment. However, they did not report any significant differences in gait speed between MCI and the groups with no cognitive impairment. Weng et al. [56] also had similar findings in that while people diagnosed with dementia walk significantly slower than the group with no cognitive impairment in both single and dual tasks, such differences did not exist between MCI and the groups with no cognitive impairment. Ali et al. [57] found some dual-task protocols to be able to discriminate among all three different groups (healthy, subjective cognitive impairment, and MCI) in terms of gait velocity. In story recall and dual-task calculation, significant differences were found only between SCI and MCI (*p* < 0.05, *p* < 0.02).

Some studies revealed that dual tasks enlarged the differences between the group with no cognitive impairment and the group with MCI and/or dementia, possibly suggesting that duals tasks could be superior in identifying people with cognitive decline than single tasks. Nasicmbeni et al. [37] found that participants performing dual tasks had a significantly lower gait speed compared to single tasks (*p* < 0.01). In particular, the percentage differences in gait speeds between groups with no cognitive impairment and groups with MCI were 19.8–23.5% across various dual tasks, which were significantly larger than the difference of 14.4% between the two groups in conducting single tasks. The larger differences between the two participant groups in dual tasks was also seen in Hunter et al. [44]. They reported that during dual tasks, people with MCI walked 16.0–28.4% (across various tasks) significantly slower than the healthy group, and the difference reduced to 8.1% in single tasks. J Lee and S Park [45] found the MCI group had significantly larger reductions in gait speed during dual tasks (27 cm/s decrease for MCI group compared to 14 cm/s decrease for control group with no cognitive impairment). The reductions were significantly lower in single tasks. While Wang et al. [55] did not report any significant differences between the MCI and healthy groups with no cognitive impairment, Weng et al. [56] found a larger difference in gait speed between people diagnosed with dementia and the group with no cognitive impairment in dual tasks compared to single tasks.

#### 3.2.2. Gait Variability

Gait variability (refer to Table 5) was another important gait parameter that displayed distinctive features in the context of discriminating between normal cognition and cognitive impairment. All of the studies [35,37,45,56] utilized the concept coefficient of variance (CoV), which is the percentage of standard deviation divided by the overall mean value. Weng et al. [56] found significantly higher variability for both MCI and dementia groups (61% and 38% increase), when compared to a control group with no cognitive impairment (34% increase) during dual-task walking. J Lee and S Park [45] also found a larger variability present in gait during dual tasks for the group with MCI compared to the group with no cognitive impairment through analysis of the coefficient of variation in stride. In particular, they found significant change in variability for the group with MCI compared to the control with no cognitive impairment (4.49 ± 1.90 in single-task condition vs. 11.21 ± 6.84 in dual-task condition, almost a 7% change in variability). Nascimbeni et al. [37] found a lower coefficient of variance (CoV) in stride time (3.17) for the MCI group, compared to the healthy group (3.58) in the single-task condition. The CoV was found to be increased to a greater extent across all dual-task conditions in the MCI group: dual tasks: 5.52, 5.42, 5.07 vs. single task 3.17), when compared to the participants with no cognitive impairment (dual tasks: 4.44,5.57, 3.93 vs. single task 3.58) and this increase is significant between single-task and all dual-task conditions (*p* < 0.01). Boripuntakul et al. [35] observed the variation in step time for groups with no cognitive impairment and groups with MCI between single- and dual-task conditions. They found significantly higher variability for MCI groups in both conditions. Further post hoc tests revealed variability differences to be significantly larger in the dual-task condition for the group with MCI compared to control groups with no cognitive impairment, compared to the single-task condition (*p* < 0.03). This further highlights the effectiveness of dual tasks in discriminating between cognitive impairment.

#### 3.2.3. Cadence and Other Spatiotemporal Parameters

Cadence was investigated in three studies [42,43,56] (refer to Table 6). Weng et al. [56] observed a significantly lower cadence in participants with dementia compared to participants with no cognitive impairment, and such differences were statistically significant throughout all different types of dual tasks. Ansai et al. [43] also found similar differences in cadence between the dementia group and the group with no cognitive impairment. However, the studies from both Weng et al. [56] and Ansai et al. [43] reported that there were no significant differences between controls with no cognitive impairment and MCI. Meanwhile, Konig et al. [42] observed a significantly lower cadence during the DT condition in both the MCI and/or dementia group, compared to the group with no cognitive impairment. Other spatiotemporal parameters including step length and width, stride time, stance, and swing time were also studied in the other reviewed studies. Boripuntakul et al. [35] found that MCI groups have a significantly lower step length (48.25 ± 5.66 cm) and lower step width (50.65 ± 5.40 cm) compared to the group with no cognitive impairment (50.15 ± 6.55 cm and 52.15 ± 6.11 cm). These parameters reduced during dual-task conditions in both MCI (step length 45.39 ± 6.88 cm and step width 48.37 ± 6.49 cm) and the controls with no cognitive impairment (47.76 ± 6.68 cm and 50.16 ± 6.32 cm). Nascimbeni et al. [37] found a longer stride time (1.21 s vs. 1.14 s) among people with MCI. Analyzing the mid-stance and swing time, Du S et al. [54] found no significant differences between groups with no cognitive impairment and groups with MCI in the ST condition. However, during dual-task conditions, they reported the MCI group had a significantly lower swing time and higher mid-stance variability compared to the group with no cognitive impairment.

Based on the results discussed in Section 3.2.1, Section 3.2.2 and Section 3.2.3, gait speed was found to be the most prominently sensitive out of the three gait parameters. There is substantial evidence of significant gait changes during dual tasking for the cognitive-impaired and dementia group not only in this systematic review, but in other experimental studies as well (more information and references available in Section 4.1 of the discussion). The number of studies that found gait speed to have significant or notable differences were also higher compared to the number of studies that found gait variability, cadence, and other gait parameters to have significant or notable differences. This indicates a strong effect for gait speed in terms of inducing change. Cadence had the lowest sensitivity in terms of effect size, as it had the fewest number of studies within the review that reported notable/significant changes in cadence across groups. However, percentage changes across the studies that did report cadence show very high variation when compared to gait speed and gait variability.

### 3.3. Secondary Task Types and Their Effect on Gait in Dual-Task Protocol

Many different types of dual-task protocols were utilized in previous studies. A backwards counting task was one of the popular secondary tasks used in six studies [35,36,37,39,42,45] involving gait tests. However, the results were mixed. Three out of these six studies [35,36,39] found that backwards counting induced a significantly larger increase in gait speed and longer stride among people with MCI compared to people with no cognitive impairment. However, Lee and Park [45] found gait speed to be significantly decreased for the MCI group by the counting. König et al. [42] reported significant changes in gait parameters for single- and dual-task conditions when using the backwards counting task and concluded that only walking speed discriminated between the control group with no cognitive impairment and the group with dementia. They did not find differences between the control group with no cognitive impairment and the group with MCI.

Meanwhile, six studies found that subtraction-based calculation consistently produced bigger differences in gait among people with and without MCI, compared to other cognitive tasks [40,44,50,52,56,57]. They noticed such secondary tasks induced a significantly larger reduction in gait velocity and an increase in postural sway among people with MCI, compared with people without cognitive impairment.

Many other secondary tasks were attempted. Ansai et al. [38,43] utilized recalling a phone number as a secondary dual task. However, they did not find any significant differences in gait that could help discriminate between older adults with no cognitive impairment and older adults with cognitive impairment. On the contrary, Ali et al. [57] found recalling a story whilst walking to be the best cognitive task for discriminating among cognitive impairment, potential cognitive decline, and normal cognition. Phonemic/verbal fluency-based secondary tasks were investigated in a few studies [37,47,48,53,56]. Such fluency-based secondary tasks included spelling letters backwards, short story recall, naming animal pictures, and word recall. Lowe et al. [47] and Wang et al. [56] observed a significant decrease in gait speed for the MCI group whilst using this sort of secondary task, whereas Nascimbeni et al. [37] found no significant differences in gait parameters between the two groups (MCI and no cognitive impairment). Ghoraani et al. [48] observed that combining machine learning with a phonemic type of task can detect up to 108 gait features during dual-task conditions, and they can help to discriminate between having no cognitive impairment and having MCI. Åberg et al. [53] observed phonemic types of tasks can help differentiate between the group with MCI and the group with no cognitive impairment, when combined with the TUG (Timed Up and Go) test as the primary task.

J Lee and S Park [45] asked participants to give priority to one of the two tasks in a dual-task condition, which means to completely focus on the prioritized task and not worry about performing the other secondary task accurately. They conducted a dual-task protocol in three different conditions: no priority (not prioritizing one task over the other, gait priority (prioritizing gait over cognitive task), and cognitive priority (prioritizing cognitive task over gait). It was observed that during both the priority conditions in the dual task, both the groups with no cognitive impairment and the groups with MCI experienced a further decrease in gait speed, with the decrease being more prominent for participants with MCI (almost 15–20 cm/s decrease compared to participants with no cognitive impairment, who only showed a decrease of 5–6 cm/s). Hunter et al. [44] used a motor task as a secondary task (carrying a glass of water). They reported a significant decrease in gait speed upon performing the secondary motor task, consistent with findings from the other studies where cognitive tasks were utilized. Meanwhile, Manicioppi et al. [49] compared three main tasks, which included foot and finger tapping and gait and used backwards counting as a secondary task. They showed that toe tapping had the highest specificity and a decent sensitivity in terms of the ability to discriminate between those older adults with MCI and those with no cognitive impairment.

Overall, various dual tasks were investigated in terms of their effect on gait indicators, particularly in discriminating between those with MCI and those with normal cognition. To summarize, backwards counting activities revealed that the MCI group had longer and wider steps. In addition, toe tapping during counting backwards showed excellent specificity and sensitivity in discriminating between having MCI and having no cognitive impairment. In addition, calculation tasks, which included serial subtraction and carrying a glass of water on a tray with one hand, resulted in a slower gait speed and more postural sway, while the Go-No/Go-task had an increased stride time and decreased number of steps. However, phonemic fluency and recalling phone numbers did not significantly improve the early detection of MCI. Considering gait speed had the most notable changes between the MCI group during dual-task scenarios and was supported by most studies, arithmetic tasks involving calculation can be deemed as one of the most effective secondary tasks for discriminating between those with MCI and those with no cognitive impairment.

### 3.4. Brain Activity During Dual-Task Protocol

Reviewing the attributes of the different brain areas as well as their activation patterns is also a source of interest in terms of understanding cognitive ability during multitasking and is a unique parameter that was investigated in four studies in this review [39,41,51,56]. These studies showcase that observing differences in brain activation patterns can indicate subtle differences during dual-task protocols, which could prove to be an independent factor in discriminating between having no cognitive impairment and when cognitive impairment is present.

These four studies in this review utilized measurements other than EEG to identify changes in brain activity during dual-task gait performance. Doi et al. [41] utilized MRI and found that dual-task gait performance was associated with a gray matter pattern of increased volume in the medial frontal gyrus, superior frontal gyrus, anterior cingulate, cingulate, precuneus, fusiform gyrus, middle occipital gyrus, inferior temporal gyrus, and middle temporal gyrus, which can help discriminate between people with MCI from the people with no cognitive impairment. Moreover, two studies [51,56] revealed that prefrontal cortex (PFC) activity in MCI measured by fNIRS increased during cognitive DT walking, which could indicate the presence of compensating mechanisms resulting from extra cognitive load. However, there were no significant effects on cognitive assessment scores, which can differentiate groups with MCI from groups with no cognitive impairment [51]. Auvinet et al. [39], through MRI assessment, focused on understanding the relationship between motor phenotypes resulting from stride performance during dual-task walking and conventional MRI findings. They found an increased hippocampal volume across phenotypes associated with stride regularity but could not replicate similar findings regarding white matter sensitivity, which is a common occurrence in MCI and/or dementia.

To summarize, changes in patterns of the brain matter volume as well as changes in brain activity distribution can be a potential marker for identifying cognitive impairment in older adults.

## 4. Discussion

The studies reviewed in this systematic review highlighted that some gait parameters have the potential to be useful tools for understanding and discriminating the presence of cognitive impairment/dementia in older adults. While analyzing gait was suggested as a tool to detect MCI and/or dementia, it should be noted that gait parameters can be influenced by many other physiological factors, such as musculoskeletal disorders and injuries. Mazaheri et al. [60] found people with ACL (anterior cruciate ligament) surgery showcased an overall slower gait speed during dual tasking. Tavakoli, Forghany, and Nester [61] observed that for people with ankle instability, plantar flexion and inversion are particularly increased. Therefore, musculoskeletal injuries contribute towards changing particular mechanics of the gait. Enlarging the differences and allowing more parameters to be significantly different between the two participant groups will increase the sensitivity of detecting cognitive decline. Adding a secondary task to gait (dual tasks) could address these challenges.

### 4.1. Single- and Dual-Task Effects on Gait Parameters

From Table 4, Table 5 and Table 6 in the results section, it is evident that even in the absence of dual tasks (single-task condition), there are notable differences in gait parameters between healthy controls, MCI, and dementia. These include a lower velocity and cadence, and higher gait variability among people with MCI and dementia. However, the differences are more prominent in the dual-task condition, as is reflected in the results section from Section 3.2.1, Section 3.2.2 and Section 3.2.3. Performing a cognitive task concurrently whilst walking requires greater attentional resources [62]. Nine studies included in this review [35,37,42,43,44,45,55,56,57] found dual tasks significantly reduced gait parameters including velocity and cadence as well as variability in spatiotemporal gait parameters, warranting the effectiveness of a dual-task protocol in inducing negative changes in gait performance, which can act as an indicator that cognition is impacted. These significant changes also suggest the potential for using a dual-task protocol to discriminate between intact cognitive ability and MCI. Seven of these nine studies [37,42,44,45,55,56,57] reviewed demonstrated the linkage between gait speeds and cognitive impairment using a dual-task protocol, which align with studies in other similar areas [63,64] reporting significant reductions in gait velocity for participants with MCI and/or dementia during a dual task. Yang et al. [65] completed a meta-analysis review and also found gait speed reduction during a dual task as a prominent parameter that can signify differences between cognitive impairment and expected cognition in a dual-task condition. Gait variability increases were also observed when compared between control groups with no cognitive impairment and groups with MCI and/or dementia during a dual-task protocol in some of the reviewed studies, and this is further corroborated in other studies showing high gait variability during dual-task walking. Increased variability in stride time [66], step velocity [67], step length, and swing time [68] was found in the gait of people with MCI and/or dementia. These changes in gait parameters for the group with cognitive impairment could be explained through utilizing the Compensation-Related Utilization of Neural Circuits Hypothesis (CRUNCH) model, which specifies that the recruitment of neural networks from both hemispheres is commonly observed among older adults and younger adults with cognitive impairment, for the purpose of recompensing the decline of executive functional abilities [69]. As a result, most older adults with MCI and/or dementia tend to slow down and alter their gait significantly as a compensatory mechanism for achieving stability during dual-task walking.

### 4.2. Effects of Secondary Task Type in Dual-Task Protocol on Gait

The secondary task type within the dual-task protocol could also have an effect in terms of the sensitivity of the changes within the gait parameters, and how accurately they might be able to discriminate between the presence of cognitive impairment and no cognitive impairment. Although Kuo et al. [51] suggested both motor–cognitive and motor–motor dual-task protocols to be effective in terms of training and improving dual-tasking capabilities, Zheng et al. [52] found motor–cognitive dual-task protocols to be more efficient and accurate in terms of identifying cognitive impairment than motor–motor dual tasks. Similarly, Hunter et al. [44] found complex cognitive tasks such as serial subtraction to be a more appropriate method for distinguishing between the presence of MCI and no cognitive impairment, rather than a motor task as a secondary task within the dual-task protocol, which did not showcase any significant differences or defining factors that can help identify cognitive impairment. This highlights the fact that certain cognitive tasks could be more challenging for cognitive processing ability and can cause more disruptions in the overall dual-task performance. Within this review, Du et al. [54] and Aberg et al. [53] unanimously agreed that word recall is a very effective cognitive task for facilitating difficulties in the dual-task protocol, as it puts more strain on working memory, which is significantly associated with straining gait parameters such as gait speed [35]. Ali et al. [57] also agreed in line with these two studies and further established that compared to random words, recalling a story provided a more significant challenge for people with cognitive impairment, when compared to people with no cognitive impairment. Arithmetic tasks, such as counting backwards, and memory tests, such as remembering animal names and so on, cause a gradual decrease in gait parameters during dual-task walking [70,71]. However, the study undertaken by Nascimbeni et al. [37] where verbal fluency-/phonemic-based dual-task protocols were used contradicts this, as they did not find significant differences. Task prioritization could be another inducer of variability in dual-task walking performance. Yogev-Seligmann et al. [72] observed that task prioritization in dual-task walking affected gait speed more prominently in younger adults compared to older adults. They found that although gait variability is impacted by dual-task performance prominently among older adults, there was no significant effect of task prioritization on variability. However, Oh-Park et al. [73] found a decreased ability in older adults to focus on motor tasks (carrying tray) while walking, which indicates that there is a tendency among older adults towards focusing on/prioritizing walking in motor-based dual-task situations. Mazaheri et al. [25] concluded that older adults tend to prioritize the cognitively challenging task over the motor task, which could lead to a high risk of experiencing a fall. These studies are in conjunction with the findings from Lee J. and S. Park [45], who found increased changes in gait velocity between MCI and groups with no cognitive impairment during both priority conditions compared to the no priority condition (almost 10 cm/s decrease in average between groups with no cognitive impairment and groups with MCI), further showcasing the effects of task prioritization. In addition to gait, toe tapping is a less complex movement that can induce major changes, which clarifies the cognitive situation during a dual-task protocol and can potentially help to discriminate between control groups with no cognitive impairment and groups with MCI and/or dementia [49].

### 4.3. Differences in Gait Performance for MCI and Dementia

This review found inconsistent findings regarding the gait differences between groups with MCI and groups with no cognitive impairment. It was observed that three out of seven studies that investigated gait speed [35,48,49] reported no significant differences between MCI and the controls with no cognitive impairment, but the remaining four studies revealed the opposite [37,44,45,57]. Variability within people with MCI could explain the inconsistent findings. In some cases, MCI is the initial stage of development towards dementia. But in some other cases, there are other situations, such as diabetes, drugs, and depression, that cause MCI. MCI does not always lead to dementia. MCI symptoms are usually mild and, in most cases, if dementia occurs, it results in gradual increases in cognitive impairment after the MCI stage. Meanwhile, these inconsistent results can also be caused by different types of secondary tasks used in the dual-task protocol. For example, Konig et al. [42] utilized backwards counting as the secondary cognitive task in the dual-task protocol, which is a measure of short-term memory for adults [74]. As people with MCI are more prone to short-term memory loss [75], their dual-task gait performance could be more impacted. Ansai et al. [43] found higher differences in cadence for dementia patients when compared to the healthy controls and MCI, and the differences between the healthy controls and dementia were significant (please see Table 6). Similar findings were also seen for cadence in the study by Wang et al. [55].

### 4.4. Brain Activity During Dual Task for MCI and Dementia

Other than gait parameters, brain activities and activation patterns were also considered as important biomarkers in this review for identifying cognitive impairment. Kuo et al. [43] suggested that older adults might have higher activation in the prefrontal cortex, which could result in gait prioritization, and findings from similar studies [28]. This is further supported by Protzak and Gramann [29], who investigated the modulations of the alpha and beta band using EEG during motor dual-task walking and found that alpha and beta desynchronization was less among older adults compared to younger adults. This lesser reduction in beta desynchronization was associated with a slower gait speed while performing dual-task walking, which could indicate an imbalance in cognitive resource allocation among older adults. There may be differences between people with neurodegenerative diseases such as Parkinson’s and dementia with cognitive impairment. Possti et al. [76] observed brain spectral power among three groups: younger adults with no cognitive impairment, older adults with no cognitive impairment, and people with Parkinson’s disease. They found similar results in terms of decreased spectral power for alpha and beta for younger adults and older adults, but people with Parkinson’s showed an increase, indicating cognitive impairment might not provoke cognitive resource allocation the exact same way as it does when there is no cognitive impairment. Kahya et al. [77] also found decreased alpha activity during a dual task for older adults with MCI, although this study did not look at gait but focused on posture whilst standing and performing a cognitive task (serial subtraction). These significant differences can be a major discriminating factor between unimpaired and impaired cognition. A similar increase in activation was also observed by Weng et al. [56] for participants with cognitive impairment, which further increases the credibility of the other studies.

### 4.5. Limitations and Final Remarks

One notable limitation observed in the studies reviewed was that there were a minimal number of studies (only 1, Ali et al. [57]) analyzing kinematic and kinetic parameters such as joint angles, joint moments, ground reaction forces, etc. All the other studies purely focused on the spatiotemporal parameters of the gait, with some additionally looking at brain activity to understand cognitive ability. Certain kinematic parameters such as hip and knee flexion and extension and ankle plantar and dorsiflexion are influential for understanding gait stability [78,79]. Investigating these kinematic parameters can possibly lead to new possible diagnostic criteria and methods, which can potentially improve the overall diagnostic capabilities of MCI and dementia. There was also a lack of studies attempting to discriminate possible differences among different types of dementia (e.g., vascular dementia, alcohol-induced dementia). In addition, dementia sometimes develops early. Although uncommon, dementia can appear in people of younger ages. Future studies should investigate if movement analysis can help identify the types of dementia and any differences between younger and older onsets of dementia. Meanwhile, all the reviewed studies did not appear to include people from First Nation or indigenous communities, which have different definitions of old age. This systematic review also had its own limitations. There were no inclusions of the gray literature, and further adjustments could be made within the search criteria to include types of sensor modalities and measurement methods. This can help to understand the effect of different experimental methods and whether they influence the quality of the results to a certain degree. Furthermore, this systematic review did not include particular effects that could happen due to different types of dementia (vascular dementia, alcohol-based dementia, dementia resulting from stroke) and just reviewed the overall effects of average dementia. Further research should be conducted to include how different types of dementia can have different outcomes in their gait measurements during dual tasking, which could potentially provide more valuable insight. Overall, the studies reviewed in this systematic review highlight gait speed, cadence, and gait variability as effective parameters for distinguishing between preserved cognition and impaired cognition (Section 4.1). They also highlighted that the type of secondary task during dual-task protocols can be sensitive in terms of distinguishing between levels of cognitive ability, particularly when comparing between different levels of cognitive impairment (MCI and dementia) (Section 4.2 and Section 4.3). Furthermore, this systematic review quantified these changes not only in terms of walking parameters, but also with changes happening within the cognitive process through looking at power and matter volume across the brain (Section 4.4). There have been previous studies investigating the potential of dual-task protocols for identifying cognitive impairment and improving gait. Ramírez, F. and M. Gutiérrez et al. [80] found a dual-task protocol effectively discriminated between cognitive impairment and no cognitive impairment as it induced a larger change within gait for those with MCI compared to those without. Pereira Oliva et al. [81] found dual-task training was a useful protocol for improving the physical movement abilities of people with neurodegenerative conditions impacting cognitive ability. However, these two studies majorly focused on analyzing movement alone and how training improved movement ability. In this systematic review, focus was provided on analyzing the changes in gait parameters across different cognition groups, and whether there were differences in these parameters that were significantly prominent enough to distinguish between impaired and preserved cognition somewhat accurately.

## 5. Conclusions

This review explored the ability of analyzing gait in both single-task (gait only) and dual-task (gait and a secondary task concurrently) protocols in differentiating between people with and without MCI and/or dementia, potentially as an alternative way to diagnose cognitive decline. A total of 23 studies, published between 2014 and 2024, were included in this review paper. This review found that changes in gait parameters such as speed, cadence, and gait variability can discriminate between people with cognitive impairment and those with no cognitive impairment. Gait velocity was consistently shown in six studies to be able to differentiate between people with and without a diagnosis of dementia. However, the results were inconsistent when comparison was made between the MCI group and the group with no cognitive impairment. The differences in gait parameters between the group with no cognitive impairment and people with MCI and/or dementia were bigger in dual tasks compared to single tasks. The type of dual task while walking can impact the difference in walking between the two participant groups. A subtraction-based arithmetic cognitive task was found to be a useful secondary cognitive task in terms of inducing changes in gait, which extensively aided in discriminating between MCI and non-MCI. Brain activity during dual-task walking (spectral power for alpha and beta, white and gray matter volume) in people with neurodegenerative disease and cognitive impairment show different patterns compared to control participants with no cognitive impairment. To conclude, analyzing the walking pattern and brain activity during a dual task can identify cognitive impairment and differentiate it from normal cognition. Further work should include emphasizing the investigation of kinematic parameters along with spatiotemporal parameters and brain activity to understand further the potential subtle differences between people with no cognitive impairment and people diagnosed with MCI and/or dementia.

## Figures and Tables

**Table 1:** Levels of evidence (Oxford Center for Evidence-based Medicine—March 2009) [34].

Level	Therapy/Prevention, Etiology/Harm
1a	Systematic review (with homogeneity) of randomized controlled trials
1b	Individual randomized controlled trial (with narrow confidence interval)
1c	All or none
2a	Systematic review (with homogeneity) of cohort studies
2b	Individual cohort study (including low-quality randomized controlled trial, e.g., <80% follow-up)
2c	“Outcomes” research; ecological studies
3a	Systematic review (with homogeneity) of case–control studies
3b	Individual case–control study
4	Case series (and poor-quality cohort and case control studies)
5	Expert opinion without explicit critical appraisal, or based on physiology, bench research, or “first principles”

**Table 2:** Grades of recommendation (Oxford Center for Evidence-based Medicine—March 2009 [34]).

Grade	Contents
A	Consistent level 1 studies
B	Consistent level 2 or 3 studies or extrapolations from level 1 studies
C	Level 4 studies or extrapolations from level 2 or 3 studies
D	Level 5 evidence or troublingly inconsistent or inconclusive studies of any level

**Table 3:** Table summarizing the main findings from the reviewed studies and their evidence level and recommendation.

Study	Evidence Level & Recommendation	Main Findings
Boripuntakul et al. (2014) [35]	2b—Individual Cohort Study, Recommendation Level—B	− Variability in different walking parameters (step length, step time, swing time, etc.) was significantly larger for MCI group. − MCI individuals had reduced balance control when undertaking challenging walking task.
Doi et al. (2014) [36]	2b—Individual Cohort Study, Recommendation Level—B	− Significant associations found between gait speed and different cognitive functions. − Working memory was significantly associated with gait speed during dual task, and visual memory was associated with gait speed during both single and dual tasks.
Nascimbeni et al. (2015) [37]	3b—Case–Control Study, Recommendation Level—B	− Both MCI and healthy control group experienced significant gait disruption during dual task. − Counting backwards during dual task worsened, while short story recall improved. − Dual-task paradigm does not improve early detection of MCI.
Ansai et al. (2017) [38]	3b—Case–Control Study, Recommendation Level—B	− AD group performed tasks significantly worse in DT condition. − Nothing to help distinguish between MCI and preserved cognition.
Auvinet et al. (2017) [39]	2b—Individual Cohort Study, Recommendation Level—B	− Dual-task cost was found to be specific for each variable and increased significantly from stride frequency to stride regularity passing by walking speed.
Crockett et al. (2017) [40]	2b—Individual Cohort Study, Recommendation Level—B	− Greater resting state DMN functional connectivity may be an underlying neural mechanism for reduced dual-task ability, slower gait speed, and greater postural sway in older adults with MCI, leading to an increased risk of mobility disability and falling.
Doi et al. (2017) [41]	2b—Individual Cohort Study, Recommendation Level—B	− Increased dual-task gait speed was associated with increased gray matter volume.
König et al. (2017) [42]	3b—Case–Control Study, Recommendation Level—B	− Gait parameters had minimal differences between healthy controls and MCI. − Between healthy controls and AD, the differences were more pronounced.
Ansai et al. (2018) [43]	3b—Case–Control Study, Recommendation Level—B	− Specific cognitive domains were associated with gait and dual-task performances in adults with normal cognition, MCI, and AD.
Hunter et al. (2018) [44]	3b—Case–Control Study, Recommendation Level—B	− Complex cognitive task (serial seven subtraction) reduced gait velocity and had more impact on MCI group compared to healthy. − Dual motor task did not show significant difference in cognitive cost.
Lee J. and S. Park, (2018) [45]	3b—Case–Control Study, Recommendation Level—B	− MCI patients had significantly slower walking speed compared to cognitively normal older adults when not prioritizing a task. − MCI shows three times more gait variability in no priority condition.
de Oliveira Silva et al. (2020) [46]	3b—Case–Control Study, Recommendation Level—B	− Cognitive task and cognitive–motor tasks showed significant differences. − Different stages of IADL showed the worst performance on tests combining motor and cognitive demand.
Lowe et al. (2020) [47]	2b—Individual Cohort Study, Recommendation Level—B	− Cognitive variables significantly predicted fluency in dual task. − Degree of physical comorbidity, level of social interaction, and age can be used as predictors of dual-task performance in cognitive–motor tasks.
Ghoraani et al. (2021) [48]	3b—Case–Control Study, Recommendation Level—B	− Gait variability was most significant in detecting cognitive decline. − Discriminating between MCI and AD more challenging using only gait features.
Mancioppi et al. (2021) [49]	3b—Case–Control Study, Recommendation Level—B	− Toe tapping better at distinguishing cognitive impairment than finger tapping. − Toe tapping showed further high specificity and decent sensitivity compared to gait and finger tapping when discriminating between MCI and healthy cognition.
Wu et al. (2021) [50]	2b—Individual Cohort Study, Recommendation Level—B	− Poor dual-task performance equals potential cognitive impairment. − Step speed and calculation speed deemed to be the most important features in dual-task-based discrimination.
Kuo et al. (2022) [51]	2b—Individual Cohort Study, Recommendation Level—B	− Cognitive dual-task training and motor dual-task training improved dual-task walking while responding to activation changes in the brain.
Zheng et al. (2022) [52]	3b—Case–Control Study, Recommendation Level—B	− Motor–cognitive dual task more likely to discriminate between MCI and healthy, rather than motor–motor dual task.
Åberg et al. (2023) [53]	2b—Individual Cohort Study, Recommendation Level—B	− Word and time are the best predictors of cognitive decline and progression towards dementia.
Du et al. (2023) [54]	3b—Case–Control Study, Recommendation Level—B	− Participants with MCI exhibit decreased swing time and terminal swing. − Subjects walked slower during dual task. − Words recall in dual task had more impact on gait regularity, velocity, and dual-task cost compared to the other tasks.
Wang et al. (2023) [55]	3b—Case–Control Study, Recommendation Level—B	− Gait speed was lower for MCI group. − Novel dual-task protocol (naming animals whilst walking). − Gait speed was significantly associated with the MoCA scores.
Weng et al. (2023) [56]	3b—Case–Control Study, Recommendation Level—B	− Individuals with mild dementia had worse walking performances for all conditions. − No significant differences in prefrontal activity among different groups.
Ali et al. (2022) [57]	3b—Case–Control Study, Recommendation Level—B	− Two different dual-task protocols (story recall and serial subtraction). − Gait speed showed significant differences under dual task for MCI and SCI (subjective cognitive impairment) − Dual task using story recall had a better sensitivity for identifying cognitive impairment.

**Table 4:** Results of gait speed in single- and dual-task scenarios (MCI = mild cognitive impairment, SCI = subjective cognitive impairment, ST = single task, DT = dual task. The ± symbol signifies the margin of error for the unit m/s).

Parameter	Study	Task Type	Population Type	Mean Parameter Values	Method of Motion Analysis	Significance
Gait Velocity	Nascimbeni et al. (2015) [37] (m/s)	Single Task	Control MCI	0.97 0.83	STEP 32, DEM Italia, Leinì, Turin, Italy using 3 footswitch sensors	Significant differences for gait velocity between ST and DT (*p* < 0.01), but not between groups
		Dual Task (phonemic fluency, short story recall, counting backwards)	Control MCI	0.78, 0.75, 0.81 0.59, 0.59, 0.65		
	Konig et al. (2017) [42] (m/s)	Single Task	Control MCI Dementia	0.88 0.77 0.75	CE marked 3D accelerometer	Significant differences for dementia when compared to the group with no cognitive impairment and the group with MCI for both conditions (*p* < 0.0001)
		Dual Task	Control MCI Dementia	0.75 0.64 0.62		
	Hunter et al. (2018) [44] (m/s)	Single Task	Control MCI	1.23 1.13	GaitRite	Significant differences found between groups in both conditions (*p* < 0.001). Greater reduction in gait speed for MCI
		Dual Task (motor, counting, motor + counting, animals, counting, motor and counting	Healthy MCI	1.21, 1.19, 1.15, 1.11, 1.02, 0.99 1.05, 1.00, 0.95, 0.88, 0.73, 0.75		
	Lee J. & S. Park (2018) [45] (m/s)	Single Task/Dual Task (no priority)	Healthy, MCI	1.05.71 ± 0.0643/0.91.97 ± 0.0887 0.9975 ± 0.1784/ 0.7286 ± 0.1845	Orthotak 3D analysis system, 6 3D cameras	Significant difference between gait priority and cognitive priority results in DT (*p* < 0.002)
		Dual Task (gait priority, cognitive priority)	Healthy MCI Healthy MCI	0.8884 ± 0.0709 0.5438 ± 0.2462 0.8771 ± 0.0788 0.5746 ± 0.1801		
	Weng et al. (2023) [56] (m/s)	Single Task	Healthy MCI Dementia	1.2068 1.1914 1.0213	GaitRite	Significant differences between control and dementia for both conditions (*p* < 0.05)
		Dual Task	Healthy MCI Dementia	1.054 0.9783 0.7984		
	Ali et al. [57] (m/s)	Single Task	Healthy (SCI) MCI	1.064 1.108 1.039	Vicon Nexus 2.8 Motion Capture System	Significant differences between control, MCI, and SCI (*p* < 0.001).
		Dual Task (serial subtraction, naming animals, story recall, words recall)	Healthy SCI MCI	0.968, 0.888, 0.844, 0.945 0.984, 0.948, 0.93, 0.974 0.865, 0.877, 0.831, 0.897		
	Wang et al. (2023) [55] (m/s)	Single Task	Healthy MCI	125.47 104.16	Stopwatch	Significant differences in speed between non-cognitive impairment and MCI, in both ST and DT condition
		Dual Task	Healthy MCI	92.8 66.31		

**Table 5:** Results of gait variability in single- and dual-task scenarios (MCI = mild cognitive impairment, ST = single task, DT = dual task. The ± symbol represents margin of error % of the base value).

Parameter	Study	Task Type	Population Type	Mean Parameter Values	Method of Motion Analysis	Significance
Gait Variability	Nascimbeni et al. (2015) (%) [37]	Single Task	Control MCI	3.58 3.17	STEP 32, DEM Italia, Leinì, Turin, Italy using 3 footswitch sensors	Significant difference between ST and DT conditions
		Dual Task (phonemic fluency, short story recall, counting backwards)	Control MCI	4.44, 5.57, 3.93 5.52, 5.42, 5.07		
	Lee J. & S. Park (2018) [45] (only focused on no priority results) (%)	Single Task	Control MCI	2.77 ± 1.05 4.49 ± 1.90	Orthotak 3D analysis system, 6 3D cameras	Statistically significant variability present between groups for dual-task condition
		Dual Task	Control MCI	2.44 ± 0.99 11.21 ± 6.84		
	Boripuntakul et al. (2014) [35] (%)	Single Task	Control MCI	10.29 ± 3.99 14.92 ± 6.87	GaitRite	Statistically significant during DT between both groups (*p* < 0.0001)
		Dual Task	Control MCI	15.46 ± 5.15 19.97 ± 8.30		
	Weng et al. (2023) [56] (%)	Single Task	Control MCI Dementia	2.44 ± 1.54, 2.59 ± 1.17 3.31 ± 3.47	GaitRite	Significant differences not found between groups (*p* > 0.05)
		Dual Task	Control MCI Dementia	3.72 ± 2.87 6.8 ± 7.65 5.38 ± 3.42		

**Table 6:** Results of cadence in single- and dual-task scenarios (MCI = mild cognitive impairment, ST = single task, DT = dual task. The ± symbol represents the margin of error in the number of steps/min).

Parameter	Study	Task Type	Population Type	Mean Parameter Values	Method of Motion Analysis	Significance
Cadence	Weng et al. (2023) [56] (Steps/min)	Single Task	Control MCI Dementia	117.78 ± 13.32 119.7 ± 8.83 108.73 ± 11.16	GaitRite	Statistically significant between control and dementia in DT condition (*p* < 0.05)
		Dual Task	Control MCI Dementia	110.02 ± 13.15 107.75 ± 12.51 98.08 ± 12.63		
	Ansai et al. (2018) [43] (Steps/min)	Single Task	Control MCI Dementia	103.2 ± 13.5 103.5 ± 14.1 100.73 ± 11	Stopwatch	Statistically significant between dementia and other groups in both conditions (*p*~0.000)
		Dual Task	Control MCI Dementia	65.8 ± 11.3 60.3 ± 11.8 53.9 ± 12.7		
	Konig et al. (2017) [42] (Steps/min)	Single Task	Control MCI, Dementia	101.57 99.95 97.19	Accelerometers	Statistically significant between ST and DT condition, but not across groups
		Dual Task	Control MCI Demetia	95.98 87.28 84.84		

## Data Availability

No new data were created or analyzed in this study.
